# EFFECTS OF AGE ON CONNECTION TO NATURE AND POSITIVE AFFECT

**DOI:** 10.1093/geroni/igz038.1029

**Published:** 2019-11-08

**Authors:** Amy Knepple Carney, Julie Patrick

**Affiliations:** 1 University of Wisconsin-Oshkosh, Oshkosh, Wisconsin, United States; 2 West Virginia University, Morgantown, West Virginia, United States

## Abstract

Socioemotional selectivity theory positis that when we feel our time as limited, when a person ages, emotion based goals become a priority (Carstensen, Isaacowitz, & Charles, 1999). Although previous studies have shown that all age groups benefit from a connection to nature (CN; Bisceglia, Perlman, Schaack, & Jenkins, 2009; Han, 2008; Mayer et al., 2009), there have been no studies conducted to determine if there are age differences in CN and how that relation contributes to positive affect. Analyses were conducted with a sample size of 152 participants with an average age of 37.55 years (SD = 15.64; Range 18 -89). Age was significantly positively associated with CN, r(151)=.16, p<.05. Additionally, an ANOVA showed that middle-aged to older adults reporting significantly higher CN than younger adults. The relation of positive affect to age and CN was then examined. In the analysis examining the effects of age and CN on positive affect, the model was significant, F(3, 146)=8.48, p<.05, R2 = .15. Both, CN, and age, uniquely contributed to the variance accounted for on positive affect, although, the interaction of CN and age did not uniquely contribute to the variance. These results may be indicative of socioemotional selectively theory, in that older adults were choosing connection to nature because it fulfilled more emotional activities/goals than the younger adults in the study. Because previous research has all but ignored the association of CN and age and their relation to positive affect, it should be considered in future research.

